# Mechanisms of DNA Methylation in Virus-Host Interaction in Hepatitis B Infection: Pathogenesis and Oncogenetic Properties

**DOI:** 10.3390/ijms22189858

**Published:** 2021-09-12

**Authors:** Dake Zhang, Shicheng Guo, Steven J. Schrodi

**Affiliations:** 1 Key Laboratory of Biomechanics and Mechanobiology, Ministry of Education, Beijing Advanced Innovation Center for Biomedical Engineering, School of Biological Science and Medical Engineering, Beihang University, Beijing 100083, China; 2 Department of Medical Genetics, University of Wisconsin-Madison, Madison, WI 53706, USA; shihcheng.guo@wisc.edu; 3 Computation and Informatics in Biology and Medicine, University of Wisconsin-Madison, Madison, WI 53706, USA

**Keywords:** HBV, epigenetics, methylation, hepatocellular carcinoma

## Abstract

Hepatitis B virus (HBV), the well-studied oncovirus that contributes to the majority of hepatocellular carcinomas (HCC) worldwide, can cause a severe inflammatory microenvironment leading to genetic and epigenetic changes in hepatocyte clones. HBV replication contributes to the regulation of DNA methyltransferase gene expression, particularly by X protein (HBx), and subsequent methylation changes may lead to abnormal transcription activation of adjacent genes and genomic instability. Undoubtedly, the altered expression of these genes has been known to cause diverse aspects of infected hepatocytes, including apoptosis, proliferation, reactive oxygen species (ROS) accumulation, and immune responses. Additionally, pollutant-induced DNA methylation changes and aberrant methylation of imprinted genes in hepatocytes also complicate the process of tumorigenesis. Meanwhile, hepatocytes also contribute to epigenetic modification of the viral genome to affect HBV replication or viral protein production. Meanwhile, methylation levels of HBV integrants and surrounding host regions also play crucial roles in their ability to produce viral proteins in affected hepatocytes. Both host and viral changes can provide novel insights into tumorigenesis, individualized responses to therapeutic intervention, disease progress, and early diagnosis. As such, DNA methylation-mediated epigenetic silencing of cancer-related genes and viral replication is a compelling therapeutic goal to reduce morbidity and mortality from liver cancer caused by chronic HBV infection. In this review, we summarize the most recent research on aberrant DNA methylation associated with HBV infection, which is involved in HCC development, and provide an outlook on the future direction of the research.

## 1. Background

Hepatocellular carcinoma (HCC) is a common human malignancy disease with a particularly high prevalence in Asia and Africa [1]. The risk factors for HCC are well documented, including hepatitis B/C virus infections, aflatoxin B exposure, and heavy consumption of tobacco and alcohol. However, its molecular pathogenesis remains poorly understood. In the past two decades, there has been great progress in the identification of candidate HCC-related genetic and epigenetic risk or disease causal factors such as *HLA*-*DRB*, *HLA-DQB1,* and perhaps other HLA regions, *PBX4*, *PNPLA3*, *GLUL,* and *STAT4* from genome-wide association studies (GWAS) [2,3,4,5,6]; genome-wide methylation differential analysis (GWMA) also have identified such examples as Ras association domain family member 1A (*RASSF1A*), *P16, SLC22A20, PNPLA7* as epigenetic factors as well as their interaction [7,8,9,10]. However, the mechanism of how the virus, as the most important risk factor, interacts with genetic and epigenetics, is still not clear.

**Figure 1:**
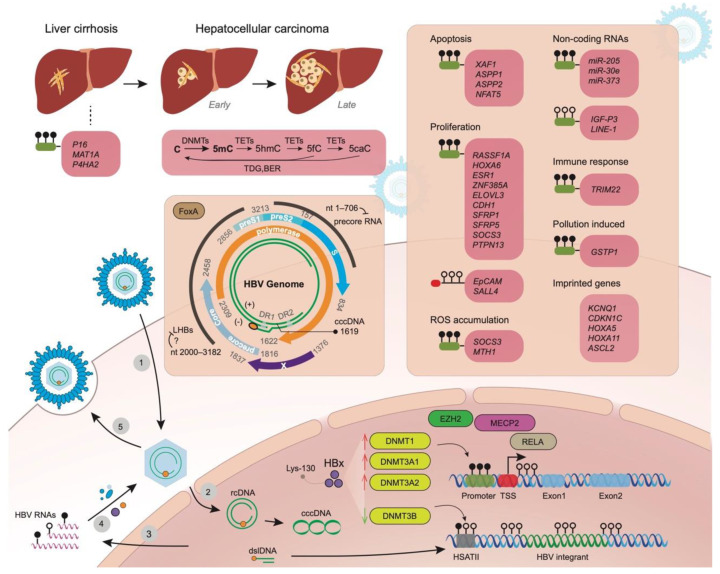
DNA methylation changes in host and viral genomes during chronic HBV infection. In the DNA methylation and demethylation cycle, DNA methyltransferases (DNMTs) catalyze the transfer of a methyl group from S-adenosyl-L-methionine to the 5th carbon atom position of cytosine (C) residues, forming 5-methylcytosine (5mC). 5mC is oxidized by ten-eleven translocation (TET) enzymes to 5-hydroxymethlcytosine (5hmC), then 5-formylcytosine (5fC), and finally 5-carboxylcytosine (5caC). Both 5fC and 5caC can be removed from DNA by thymine DNA glycosylase (TDG) in combination with base excision repair (BER) to restore an unmodified C. This process occurs along with liver disease progression, from liver cirrhosis to hepatocellular carcinoma. HBV infection can cause changes of DNMTs expression, particularly via viral X protein (HBx) whose Lys-130 may be crucial in its activity. The subsequent upregulation of *DNMT1*, *DNMT3A1,* and *DNM3A2* is believed to contribute to the promoter methylation (green rounded rectangle with black lollipops) and suppressed expression of target genes in the majority of cases, in company of host factors such as EZH2, MECP2, and RelA. Meanwhile demethylation of CpG sites downstream of the transcription start site (TSS) (red rounded rectangle with hollow lollipops) has also been observed. Meanwhile, the downregulation of *DNMT3B* can cause the demethylation of some host genomic regions, such as human satellite 2 repeat sequences (HSATII). For non-coding RNAs, both hypermethylation and hypomethylation changes in promoters are also observed. Methylation changes of host genes may result in the phenotype changes of affected hepatocytes, e.g., apoptosis, proliferation, and reactive oxygen species (ROS) accumulation; and they are also able to influence the interactions of cells with immune cells and pollutants. In addition, aberrant methylation of imprinted genes occurs during tumorigenesis. After the HBV DNA imported into the hepatocyte nucleus, it forms covalently closed circular DNA (cccDNA), serving as the template for all viral RNAs. HBx-induced methylation of C-1619 in cccDNA increases its ability of viral particle production. The human FoxA pioneer transcription factor is known to bind to HBV promoters, and its deficiency is reported to introduce increased methylation of nt 1–706 and nt 2000–182, particularly leading to decreased precore RNA synthesis. HBV replication generates double strand linear DNA (dslDNA), which can be incorporated into the host genome and its subsequent methylation level seems to be consistent with the surrounding regions at the integration sites.

DNA methyltransferases (DNMTs) turn cytosine (C) into 5-methylcytosine (5mC) and ten-eleven translocation (TET) enzymes oxidize 5mC into 5-hydroxymethlcytosine (5hmC), 5-formylcytosine (5fC), and 5-carboxylcytosine (5caC) sequentially, the last two of which can be restored to an unmodified C (Figure 1). By far, 5mC is the most well studied and almost all methylation changes here in the review are referred to 5mC. Different viral hepatitis may cause distinct epigenetic changes within the host genome. As observed by Shih et al. (2016), increased methylation of Paired-box 6 (*PAX6*) was more frequently seen in HCV-positive HCC (61.3%) in comparison with HBV-positive HCC (22.1%) in a cohort of 160 patients [11]. The promoter methylation is thought to account for the lack of *RB1* expression in both HBV and HCV-associated HCC [12]. Meanwhile, certain methylation changes may be common in HCC development and are not specific to viral infection. Li et al. (2018) reported the promotor of *TMEM176A*, an activator of *ERK* signaling, was methylated in 75.4% of HCC, which was not associated with HBV infection status [13]. The differentially-methylated CpG sites were enriched in enhancers, promoters, CpG islands, and surrounding regions [14]. To provide a better understanding of the interaction role of HBV/HCV with genetic and epigenetic factors, we performed a comprehensive review of the most recent findings between virus and DNA methylation at different molecule regulation levels.

## 2. Aberrant Methylation of Promoter Regions of Genes in Liver Disease Progression Related to Tumorigenesis

DNA methylation changes are a feature of tumorigenesis, and one important mechanism by which hepatocytes respond to HBV infection is through upregulation of DNA methyltransferase genes, which may contribute to the increased tumor risk during chronic infection [15]. Liu et al. (2019) measured DNA methylation in HBV-related HCC and paired normal tissues, and found HBV infection corresponded to (i) 5mC, 5hmC, or 5fC decreases at the early stage of HCC (Figure 1) [16], (ii) was associated with decreased TET enzyme activity, and (iii) partial or complete reduction in DNA methylation-related enzymes. Although global hypomethylation is a feature of tumor genomes, hypermethylated sites can differentiate particular tumors from normal tissues.

Numerous studies have shown that the HBV-encoded X protein (HBx) is heavily involved in interactions with host gene networks and impacts HBV-mediated HCC progression. Park et al. (2007) comprehensively evaluated the HBx expression on host epigenetic changes, and found increased total DNMT activity, up-regulation of *DNMT1*, *DNMT3A1*, and *DNMT3A2*, as well as selectively promoted regional hypermethylation of specific tumor suppressor genes [17]. HBx specifically repressed insulin-like growth factor-3 (*IGF3*) expression through de novo methylation via *DNMT3A1* and *DNMT3A2* and by inhibiting SP1 binding via recruiting methyl CpG binding protein 2 (*MECP2*) to the newly methylated SP1 binding element (Figure 1) [17]. HBx also induced global hypomethylation of human satellite 2 repeat sequences (HSATII) by down-regulating *DNMT3B* (Figure 1). The prevalence of these specific methylation abnormalities by HBx was significantly correlated with HBx expression in HBV-infected HCC patients [17]. Additionally, Huang et al. (2010) reported that HBx downregulated miR-152, and its target *DNMT1* was subsequently upregulated, leading to aberrant DNA methylation [18]. Vivekanandan et al. (2010) transfected HeG2 with full-length HBV DNA according to the procedure designed by Gunther et al. [19,20]. They observed that *DNMT2* activity had the greatest increase of DNMTs [19]. Benhenda et al. (2013) reported that the upregulation of DNMTs was seen within 2–4 days after transfection, indicating a quick response of infected cells [21]. Intriguingly, they also found that fresh medium alone may also induce the upregulation of DNMT activity of the cell lines, strongly suggesting that care should be taken in interpreting results from functional experiments evaluating DNMT induction [21]. To observe the changes in host CpG sites, they collected DNA samples after 15 days of infection and applied a microarray to screen for these changes. Interestingly, they also reported that the HBV DNA fragments without replication activity could induce methylation changes in the host genome.

Promoter methylation changes of diverse genes are associated with liver disease progression. Generally, HBV infection causes promoter hypermethylation to suppress gene activity. Su et al. (2007) compared the promoter methylation in p14 (ARF), p16 (INK4a), O(6)-methylguanine-DNA methyltransferase (MGMT), glutathione S-transferase pi (GSTP1), and E-cadherin (E-Cad, also *CDH1*) in noncirrhotic, cirrhotic, and HCC tissues; p16(INK4a) promoter methylation increased along with liver disease progression; *GSTP1* promoter hypermethylation occurred more frequently in HBV related HCC; while p16(INK4a), MGMT, and p14(ARF) promoter hypermethylation did not show this tendency [22]. P16 hypermethylation may occur early before HCC development, and Shim et al. (2003) found that P16 hypermethylation increased from cirrhotic nodules through dysplastic nodules [23]. Kuramoto et al. (2017) investigated the methylation alterations in non-cancerous liver tissue, showing NASH had featured DNA methylation changes with good confidence to discern them from lesions of cirrhosis or viral hepatitis; further, aberrant methylation identified in NASH-related HCC were inherited from the non-cancerous liver tissue showing NASH—a finding which was aggravated with disease progression [24]. Methionine metabolism is known to occur in human liver cirrhosis, and Avila et al. (2000) showed that promoter hypermethylation of methionine adenosyltransferase (*MAT1A*) could contribute to its decline in cirrhosis [25]. Bing et al. (2014) further showed HBx could recruit *DNMT1* causing the promoter hypermethylation and inhibiting the glucocorticoid receptor (GR) to the glucocorticoid-response element (GRE) of the *MAT1A* promoter [26]. *ZNF382* was frequently downregulated by promoter methylation in HBV-related HCCs relative to HBV-infected liver cirrhosis tissues and decreased expression of *ZNF382* was strongly correlated with poor survival in early-stage HCC patients [27]. Nevertheless, not all abnormal gene expression is due to aberrant promoter methylation. Qiu et al. (2013) reported the expression of DNMTs induced by HBx failed to change the methylation status of the *PDCD4* promoter, and the deregulation of *PDCD4* transcription in hepatocarcinogenesis was due to HBx-mediated miR-21 upregulation [28].

In some cases, HBx-introduced promoter hypermethylation happens to miRNAs, and it removes the suppression of miRNAs on their target genes. Zhang et al. (2013) reported that although miR-205 targets HBx mRNA, HBx was able to induce hypermethylation of the miR-205 promoter and abrogate its effect on tumor suppression in carcinogenesis [29]. Feng et al. (2017) illustrated that HBx suppressed miR-30e expression by inducing the methylation of its promoter, possibly mediated by EZH2-formed complexes [30]. The subsequent upregulation of prolyl 4-hydroxylase subunit α2 (P4HA2), as a crucial enzyme catalyzing collagen formation, increased the collagen deposition in the liver in vivo and in vitro, promoting liver fibrosis and tumorigenesis [30].

The mechanism of promoter methylation of *E-Cad* has been thoroughly studied. As a tumor suppressor, *E-Cad* under-expression was one feature of a subgroup of HCC (G6) identified by Boyault et al. (2007) in their transcriptome classification of HCC [31]. For HBsAg-positive patients, *E-Cad* promoter hypermethylation remained high in both nontumorous tissues and HCCs, while it was lower in HCCs than in nontumorous tissues of HBsAg-negative patients [22]. Wei et al. (2002) suggested the HBV infection may lead to hypermethylation of the promoter region of *E-Cad*, whose expression is frequently lost in HCC [32]. Lee et al. (2005) demonstrated that this methylation change accounting for the HBx and the Lys-130 in its transactivation domain is essential. They also showed HBx induces *DNMT1* expression by stimulating its transcription [33]. Liu et al. (2006) also showed the HBx is responsible for the hypermethylation of the *E-Cad* promoter and further pointed out that decreased expression of *E-Cad* was also associated with beta-catenin accumulation in the cytoplasm and/or nuclei, resulting in increased cell migration contributing to hepatocarcinogenesis [34]. In addition to promoter hypermethylation of *E-Cad*, Arzumanyan et al. (2012) further proposed that HBx may recruit the mSin3A/histone deacetylase complex to the Snail-binding sites in human *CDH1* and cause its histone deacetylation [35]. They also pointed out that miR-373, upregulating *E-Cad* expression, was also downregulated by HBx, which may also explain the loss of E-cadherin in HBV-related carcinogenesis [35].

## 3. Apoptosis, Proliferation, and Reactive Oxygen Species (ROS) Accumulation

Efforts have been made to reveal the contribution of DNA methylation changes underlying distinct biological processes. It is known that HBV infection inhibits apoptosis by interfering with multiple signaling pathways. Zhang et al. (2008) showed that in hepatoma cell lines, promoter methylation was responsible for the low expression of X-linked inhibitor of apoptosis protein-associated factor 1 (*XAF1*) [36]. The *XAF1* promoter methylation occurred in 66% of the HCC samples they collected, and low expression of *XAF1* resulted in a reduction in recurrence-free survival of patients by over one year [36]. The promoter hypermethylation s contributed to lower expression of *ASPP1* and *ASPP2* in tumor tissues, suppressing their regulation of apoptosis through interaction with p53 and its family members. However, only the methylation of *ASPP2* may be induced by HBx expression [37]. Qin et al. (2017) illustrated that HBV induced hypermethylation of the AP1-binding site in the *NFAT5* promoter in hepatoma cells, and suppression of *NFAT5* may lead to reduced apoptosis of hepatoma cells [38].

During chronic infection, methylation changes can be observed for genes influencing cell proliferation, e.g., *RASSF1A*, *Homeobox A6* (*HOXA6*), estrogen receptor 1 (*ESR1*), *zinc finger protein 385A* (*ZNF385A*), and ELOVL fatty acid elongase 3 (*ELOVL3*) [39]. According to proliferation ability, HCCs have been broadly grouped into two classes: proliferation and nonproliferation, which reflects the fundamental differences in cell division in these two HCC classes. Tumors of the proliferation class are likely to be recurrent or lead to a shorter survival time, and they demonstrate heterogeneous changes of signaling pathways related to proliferation [40]. *LAMA2* mutations are found in 5%–15% of diverse tumors, and Jhunjhunwala et al. (2014) reported that promoter hypermethylation induced low expression of *LAMA2*, which represents a highly recurrent and proliferative HCC [41]. Xie et al. (2014) reported that HBx can induce the hypermethylation of Secreted frizzled-related protein 1 and 5 (*SFRP1* and *SFRP5*), and epigenetic silencing of these antagonists of the Wnt signaling pathway leads to the pathway activation which subsequently promotes cell growth and HBx induced epithelial–mesenchymal transition [42]. Zhang et al. (2015) demonstrated the hypermethylation of the CpG island close to cytokine signaling 3 (*SOCS3*) TSS accounted for its suppression in HCC losing its inhibition role on proliferation [43]. HCC had the promoter region of *TMEM176A* frequently methylated independent of HBV infection (Li et al., 2018), whose protein product suppresses HCC growth by inhibiting the *ERK* signaling pathway [13]. Yang et al. (2019) discovered that HBx may induce the methylation of CpG islands of miR-18 and subsequently inhibit its expression, and the suppression of miR-18 induced the nucleolar spindle-associated protein 1 (*NUSAP1*) upregulation which promotes the proliferation of hepatoma cells [44]. In hepatocytes. polycomb repressive complex 2 (*PRC2*) silences the EpCAM expression, which upregulates *c-myc* and induces cell proliferation [45]. Fan H et al. (2016) showed that this suppression can be removed by HBx induced demethylation of the CpG dinucleotide located adjacent to NF-κB/RelA half-site downstream from EpCAM transcriptional start site [46]. By chromatin immunoprecipitation assays, they further showed that RelA forms a complex with *EZH2*, *TET2,* and *DNMT3L* in the presence of HBx, and proposed the binding of RelA to the half-site may direct TET2 to demethylate the nearby CpG sites [46]. HBV infection may contribute to the re-expression of Sal-like protein 4 (*SALL4*), an embryonic stem cell transcriptional regulator, in HCC. Fan et al. (2017) pointed out that HBV-related HCC and HBV replicating cells had demethylation of specific CpG sites downstream of *SALL4* TSS, which influences *SALL4* transcriptional elongation [47]. They further showed that these demethylated CpGs are within binding sites of octamer-binding transcription factor 4 (*OCT4*) and signal transducer and activator of transcription3 (*STAT3*), which interact with embryonic stem cell BAF (esBAF) complex and recruit BRG1, a subunit of the Brg1/BAF chromatin-remodeling complex [47]. In particular, *OCT4* is repressed by *PRC2* [48], and HBx may account for the loss of *PRC2* function in HBV-related HCC [46,49,50,51,52]. Yan et al. (2020) report that protein phosphatase nonreceptor type 13 (*PTPN13*) expression was significantly lower in HBV-positive hepatocellular carcinoma tissues. Patients with low *PTPN13* expression and an HBV-positive history had the poorest overall survival. *PTPN13* downregulation induced by HBx-induced promoter methylation promoted HCC proliferation due to increased c-Myc mRNA [53].

Tumors are known to have increased ROS production, which can activate pro-tumorigenic signaling and enhance cell survival and proliferation [54]. Roles of ROS in HBV-related tumorigenesis and DNA methylation changes have also been explored recently. HBV-induced mitochondrial ROS accumulation upregulated Snail expression, which epigenetically suppressed *SOCS3* expression together with *DNMT1* and *HDAC1* [55]. The low expression of *SOCS3* led to the sustained activation of the IL-6/STAT3 pathway ultimately contributing to hepatocarcinogenesis [55]. High levels of ROS can induce oxidative DNA damage, which may produce oxidized nucleotides, such as 8-hydroxy-2-deoxyguanosine (8-OHdG). Nucleotide pool sanitization enzymes, including *MTH1* (*NUDT1*), *MTH2* (*NUDT15*), *MTH3* (*NUDT18*), and *NUDT5*, prevent oxidized nucleotides from incorporation into DNA. Lin et al. (2018) observed that in vitro and in vivo HBV caused an increase of 8-OHdG within infected hepatocytes due to reduced expression of *MTH1*, whose promoter activity was inhibited by HBx induced hypermethylation [56].

## 4. Hypomethylation, Methylation Changes to Non-Coding Genes Other Than miRNAs and Imprinted Genes

In the aforementioned promoter methylation changes of miRNAs, HBx commonly introduces promoter hypermethylation and inhibits miRNA expression. However, it is not always the case for all non-coding RNAs. Tang et al. (2015) speculated that the overexpression of transcripts from P3 and P4 promoters of the *IGF2* gene may be driven by HBx and showed in cell lines that HBx induced hypomethylation of P3 promoter and improved IGF2-P3 transcript expression [57]. Gao et al. (2014) claimed nearly 90% HCC showed hypomethylation of LINE-1 promoter compared with HBV-related cirrhosis and normal controls, and LINE-1 promoter hypomethylation accounted for the significantly reduced survival time of patients (e.g., overall survival post-resection of a median of 22 months in comparison with ~60 months of patients without this change) [58].

Imprinted genes are mono-allelically expressed in a parent-of-origin-dependent manner and methylation is the crucial modification to sustain their status. It is suggested that their methylation status is susceptible to deregulation in cancer considering genome-wide epigenetic changes as well-known tumor genomic features. Lambert et al. (2015) demonstrated the aberrant methylation process during liver disease progression was more likely to affect the imprinted genes and found HCC was likely to have hypomethylation of 15q11–13, which is the imprinting control region harboring the cluster of imprinted genes [59]. They further pointed out that methylation changes of paternally expressed genes may be more susceptible to epigenetic disruption in carcinogenesis, such as *KCNQ1*, *CDKN1C*, *HOXA5*, *HOXA11*, and *ASCL2* [59].

## 5. Changes of Host Immune Status, Infected Hepatocytes, and Exposure to Environmental Pollutant

The CpG sites in PBMC (peripheral blood mononuclear cell) DNA may have their quantitative state of methylation correlated with the liver disease progression in patients with HBV infection. Recently, Li et al. (2020) identified 7888 sites of this type in a cohort of 48 HBV-related liver disease patients and claimed their methylation level reflected the epigenetic reprogramming of the immune and inflammatory responses during chronic infection [60]. Peripheral blood DNA methylation level is used to calculate the epigenetic age (Horvath clock), and Gindin et al. (2020) reported the inconsistency between epigenetic age and chronological age in patients with HBV and HCV infection. They proposed that accelerated epigenetic age is a feature of viral hepatitis, which can be slowed down by antiviral treatment [61]. Okamoto et al. (2014) also presented the time-dependent manner of methylation changes during HBV infection in hepatocytes [39]. Nevertheless, unlike HBV, they found HCV infection did not induce aberrant methylation in HCC cell lines, which inspired them to identify the involved immune factors. They proposed the natural killer cell activity via IFN (interferon)-gamma as the major cause of these aforementioned DNA methylations [39]. The superfamily of tripartite motif-containing (TRIM) proteins induces a response to IFNs that involves innate immunity and antiviral defense against a broad range of viral infections [62]. Lim et al. (2018) found the HBx-mediated suppression of TRIM22 may evade the antiviral effect of IFNs, and further showed that among the multiple CpG sites within its promotor that they examined, only the CpG (+71) had its methylation status regulated by HBx, which reduces the IFN regulatory factor-1(IRF1) binding affinity and thereby suppresses the IFN-stimulated induction of TRIM22h which impedes IFN regulatory factor-1 [63]. This may help HBV evade the host’s innate immune system. Jin et al. (2019) attributed the reduced activity of pathways in PBMCs to hypermethylation of their member genes, which can be a feature of particular phases during infection, e.g., *RASSSF1* and *cyclin-dependent kinase inhibitor* 2A (*CDKN2A*) for chronic infection [64].

Exposure to environmental pollutants can lead to methylation level changes in gene promoter regions. Glutathione S-transferases are a type of enzyme known to defend cells against diverse damage caused by oxidant and electrophilic carcinogens, and GSTP1 is the pi-class glutathione S-transferase. GSTP promoter hypermethylation and B[a]P diol epoxide-albumin (BPDE-Alb) were positively correlated with HCC incidence [65]. In HBV-associated HCC, Zhong et al. reported the common hypermethylation of GSTP1 gene and low protein expression, and the DNA methyltransferase inhibitor 5-aza-deoxycytidine reversed the hypermethylation of GSTP1 in Hep3B and HepG2 cell lines [66]. GSTP1 expression was completely depleted in HepG2.2.15 cells due to its promoter hypermethylation, and Niu et al. (2009) suggested the HBx accounted for the methylation change [67]. Lambert et al. (2011) reported the hypermethylation of RASSF1A, GSTP1, CHRNA3, and DOK1 in HCC tumors, and they also claimed that alcohol intake was related to hypomethylation of MGMT and hypermethylation of GSTP1 associated with HBV infection [68]. Recently, Tian et al. (2020) found that the concentration of environmental pollutant organochlorine pesticides (OCPs) in blood correlated with GSTP1 methylation and increased the risk of hepatocellular carcinoma [69].

## 6. Viral Genome Methylation in HBV Replication, Modifications of cccDNA and HBV Integrants

Pioneer transcription factors are critical determinants of tissue-specific gene expression and functions of the HBV genome within hepatocytes should be under their regulation [70]. Vivekanandan et al. (2008) illustrated that methylation patterns of the HBV genome differ between patients with occult and noncult infections, and the former cases had HBV CpG island 2 more densely methylated and the latter had island 1 more densely methylated [71]. Later in 2009, they also illustrated that the methylation level of viral DNA affected the protein production ability [72]. Uchiad et al. (2017) applied combined treatment of Peginterferon and Entecavir and successfully achieved a long-term loss of HBsAg in several human hepatocyte chimeric mice, and according to HBV genome analysis, they attributed the suppression of HBV activity to the G-to-A hypermutation and GpG III island methylation and speculated their antiviral effects without the requirement of cellular immune response [73]. FoxA pioneer transcription factor is known to have seven binding sites in the HBV genome and cell culture experiments show FoxA is involved in the regulation of all four HBV promoters [74]. Oropeza et al. (2020) illustrated the dynamic changes in HBV DNA methylation during neonatal liver development, balanced by FoxA-recruited Tet-mediated DNA demethylation and Dnmt-mediated DNA methylation activities [75]. McFadden et al. (2017) and colleagues showed that in FoxA-deficiency in the HBV transgenic mice producing a limited amount of FoxA3, significantly increased methylation levels were observed in nt 1–706 and nt 2000–3182 (Figure 1) [76]. The former was almost 100% methylated, leading to significantly decreased HBV 3.5 kb precore RNA synthesis; and the latter harbors the large and major surface antigen promoter and three FoxA binding sites [74,77]. This observation is consistent with the study of Nakamura et al. (2020), comparing the effects of in vitro methylation on viral protein productions in distinct genotypes. They found for genotypes B and C, the reduction of HBeAg was more than that of HBsAg [78]. Using nanopore sequencing, Goldsmith et al. also reported the relatively high methylation level in preS1/preS2 region for genotypes A and D [79].

DNA methylation is essential for the activity of covalently closed circular DNA (cccDNA), and diverse protocols have been developed to determine its patterns [80]. The cotransfection of HBV with DNMT3a showed that the methylation of cccDNA led to decreased production of both pregenomic and precore RNA, but the precore RNA decline seemed to be more significant, possibly complicated by RNA methylation [21]. Lee et al. (2019) verified that HBx could induce the methylation of C-1619 in cccDNA, which is a highly conserved nucleotide in the binding site of HBV negative regulatory element-binding protein [81]. Its methylation interrupted that binding process and led to the activation of the core promoter responsible for a four-fold increase in HBV particles compared to the situation when HBx was absent [81]. In addition, the methylation status of cccDNA may also influence the therapeutic strategies aiming at eliminating cccDNA. Kostyushev and colleagues (2019) first reported that methylated HBV cccDNA may be resistant to the degradation effect of CRISPR/Cas9-based anti-HBV therapeutics [82]. van Breugel et al. (2012) argued that the gene expression activation of HBx functions may only work for extrachromosomal DNA, rather than integrants [83]. They demonstrated its consistent activation effect on transiently transfected constructs independent of regulatory elements within them, and this effect disappeared once these constructs were incorporated into the host genome. They showed that the activity required HBx binding to the cellular UV-damaged DDB1 E3 ubiquitin ligase [83].

It has long been noticed (Miller et al., 1983) that integrated HBV DNA in PLC/PRF/5 cell line is methylated, but the region coding for viral surface protein always remains unmethylated unlike the extensively methylated region coding for core protein [84]. Bowyer et al. (1987) observed the methylation status of HBV integrants was not consistent among diverse HCC samples, among which some were hypomethylated [85]. Miyoshi et al. (1992) claimed that 5-azacytidine (5-AZA) treatment may induce hypomethylation of HBV integrants and thereby increase the HBsAg production of a hepatoblastoma-derived cell line HB611 [86]. Watanabe et al. (2015) showed in cell lines and tissues that the methylation level of HBV integrants was associated with that of the neighboring human genome, and HBx regions within integrants stayed unmethylated even when other regions all became methylated. HBV integrations seemed not to change but “follow” the methylation level of interrupted regions of the host genome [87]. Hama et al. (2018) attributed frequent detection of HBV integration sites within inactive chromatin regions in cancer cells to negative selection for integrations in active chromatin regions [88]. Nevertheless, we observed that the hypomethylation of previously reported integration sites may be a good indicator of HCC development [89]. HBV integrations are known to predispose the liver to HCC and have attracted increasing research interest, and recently their biological impacts, particularly how viral integrants complicate the damaging of hepatocytes due to their diverse ability of viral protein production, are extensively reviewed elsewhere [90,91].

## 7. Technical Challenges in Future Research

Frommer et al. combined bisulfite conversion with sequencing in 1992 [92], and it offered a viable method to measure DNA methylation through sequencing. The treatment converts cytosine residues to uracil but leaves 5-methylcytosine residues unaffected. By comparing the sequence composition of the resulting products with the reference genome, methylated sites can be identified. Since then, diverse solutions have been developed, such as methylation-specific PCR [93], methylation-sensitive single-nucleotide primer extension [94], and the reduced representation bisulfite-sequencing method [95]. In the early stage, the PCR-based detection method typically interrogates limited CpG sites, perhaps one or two CpG sites for each promoter. In recent years, detection methods for CpG sites or specific regions have gradually become the alternatives for the validation of interesting candidates. Whole-genome bisulfite sequencing (WGBS) theoretically captures all the DNA methylation changes [96], but its high cost limited the sample size in one study. Hence, microarray techniques help to profile CpG sites across the genome in relatively large sample sizes [97]. Nevertheless, the detection sensitivity of this technique may be lower than the PCR-based or sequencing solutions. Recently, nanopore sequencing has been used to directly detect the 5mC in the naïve DNA/RNA sequence without bisulfite treatment, avoiding sample loss and introduced biases during bisulfite conversion [98].

By far, the majority of genome-wide methylation analyses have been performed on bulk tissues, which are composed of diverse types of cells with an inconsistent percentage in tissue aliquot due to random sampling or individual differences. This ignores the fact that DNA methylation is cell-type specific, and the averaging of methylation levels of a specific site may obscure the biological significance contributed by a subgroup of the cell population. Overall, cell-type based highly accurate deconvolution [99,100,101] and single-cell-based DNA methylation (sc-5mC) [102] detection assays will be more powerful approaches for future DNA methylation research. Finally, causal inference based on genetic, epigenetic, and interaction will identify more disease-causing genes and therapeutic targets through the modeling of interactions between HCC and host pathways.

## Data Availability

Not applicable.
